# The Importance of Systematically Reporting and Reflecting on eHealth Development: Participatory Development Process of a Virtual Reality Application for Forensic Mental Health Care

**DOI:** 10.2196/12972

**Published:** 2019-08-19

**Authors:** Hanneke Kip, Saskia M Kelders, Yvonne H A Bouman, Lisette J E W C van Gemert-Pijnen

**Affiliations:** 1 Centre for eHealth and Wellbeing Research Department of Psychology, Health and Technology University of Twente Enschede Netherlands; 2 Department of Research Stichting Transfore Deventer Netherlands; 3 Optentia Research Focus Area North-West University Vanderbijlpark South Africa; 4 Faculty of Medical Sciences Universitair Medisch Centrum Groningen Groningen Netherlands

**Keywords:** eHealth, technology development, virtual reality, forensic psychiatry, community-based participatory research, human-centered design, case study

## Abstract

**Background:**

The use of electronic health (eHealth) technologies in practice often is lower than expected, mostly because there is no optimal fit among a technology, the characteristics of prospective users, and their context. To improve this fit, a thorough systematic development process is recommended. However, more knowledge about suitable development methods is necessary to create a tool kit that guides researchers in choosing development methods that are appropriate for their context and users. In addition, there is a need for reflection on the existing frameworks for eHealth development to be able to constantly improve them.

**Objective:**

The two main objectives of this case study were to present and reflect on the (1) methods used in the development process of a virtual reality application for forensic mental health care and (2) development model that was used: the CeHRes Roadmap (the Centre for eHealth Research Roadmap).

**Methods:**

In the development process, multiple methods were used to operationalize the first 2 phases of the CeHRes Roadmap: the contextual inquiry and value specification. To summarize the most relevant information for the goals of this study, the following information was extracted per method: (1) research goal, (2) explanation of the method used, (3) main results, (4) main conclusions, and (5) lessons learned about the method.

**Results:**

Information on 10 methods used is presented in a structured manner. These 10 methods were stakeholder identification, project team composition, focus groups, literature study, semistructured interviews, idea generation with scenarios, Web-based questionnaire, value specification, idea generation with prototyping, and a second round of interviews. The lessons learned showed that although each method added new insights to the development process, not every method appeared to be the most appropriate for each research goal.

**Conclusions:**

Reflection on the methods used pointed out that brief methods with concrete examples or scenarios fit the forensic psychiatric patients the best, among other things, because of difficulties with abstract reasoning and low motivation to invest much time in participating in research. Formulating clear research questions based on a model’s underlying principles and composing a multidisciplinary project team with prospective end users appeared to be important in this study. The research questions supported the project team in keeping the complex development processes structured and prevented tunnel vision. With regard to the CeHRes Roadmap, continuous stakeholder involvement and formative evaluations were evaluated as strong points. A suggestion to further improve the Roadmap is to explicitly integrate the use of domain-specific theories and models. To create a tool kit with a broad range of methods for eHealth development and further improve development models, studies that report and reflect on development processes in a consistent and structured manner are needed.

## Introduction

Electronic health (eHealth)—a technology to support health, well-being, and health care—can offer many benefits, such as increased quality of care, easily accessible health care, and increased self-management [1]. However, these benefits are often not fully realized in practice [2]. A possible explanation for this is that technology does not optimally fit the needs, wishes, and characteristics of the involved end users and their context [3-5]. A way to improve this fit is thorough participatory eHealth development in which potential end users are structurally involved in the development process [1,6-8]. Consequently, many efforts have been made to create models, approaches, and guidelines for development of eHealth technologies. Examples are the CeHRes Roadmap (the Centre for eHealth Research Roadmap) [9], the person-based approach [10], the accelerated creation-to-sustainment model [11], intervention mapping [12], the persuasive system design model [13], and the agile science approach [14]. Most of these models and approaches do not offer concrete prescriptions for ready-to-use research methods that fit specific contexts and people. Instead, they present abstract guidelines for development to support researchers in shaping their development process. Although a step-by-step, detailed prescription of a specific development process does not seem feasible because of different characteristics of contexts, people, and technologies, there does seem to be a need for more knowledge and guidelines on how to apply these models in practice [14]. To support researchers in operationalizing development models, we propose that a general tool kit with a broad range of eHealth development methods might be developed. Such a tool kit can provide an overview of broad-range development methods and guidelines on when and how to apply them. In this way, it can support researchers in choosing appropriate methods for the context and end users with which they are working and different phases of their development process. Using a tool kit can prevent other researchers from having to reinvent the wheel and result in more efficient and better substantiated development processes.

To create a tool kit, more generalizable knowledge on eHealth development methods is necessary. To build this knowledge base, more case studies that explain and reflect on specific development methods used seem to be necessary [15]. On top of that, there also should be more critical reflection on eHealth development models [2,10], mostly to be able to constantly improve these models to keep them in line with the most recent insights. Although there are several studies that describe development processes of eHealth technologies [16-20], there seems to be no standardized way of reporting and reflecting on the methods used. Also, an in-depth critical reflection on the development model used is often lacking. To fill these gaps in the literature, this case study presents and reflects on the development process of a virtual reality (VR) application for forensic mental health care. This study had 2 main goals. First, it aimed to increase knowledge on suitable methods for participatory eHealth development. This contributes to creating the aforementioned tool kit. Second, it aimed to reflect on the development model used to guide the process: the CeHRes Roadmap. Combined with other studies that reflect on this model, this can result in further improvement of the Roadmap.

## Methods

### The CeHRes Roadmap

In this study, the aforementioned CeHRes Roadmap [9] was applied to shape the development process of the VR application. This development model specifically focuses on eHealth development, implementation, and evaluation with structural stakeholder involvement [1,6,9]. The Roadmap has been proven useful for eHealth development in multiple settings [16,18,21] and seems to be suitable for development in complex contexts [9], such as forensic mental health care. The Roadmap is based on 5 principles that are also acknowledged by other studies on eHealth development:

- eHealth development should be a participatory process—structurally and actively involving stakeholders during development is important [7,10,12,21].
- eHealth should not be seen as a separate, stand-alone tool but has to be integrated in a health care context, which also implies changes in the way health care is delivered [5,22,23].
- eHealth development and implementation should be intertwined; implementation is a very complex activity that should be accounted for from the start of the development process [24,25].
- eHealth technologies should be based on theories from persuasive design, which can be used to support behavior and attitude change via technology [13].
- Continuous, formative evaluation in eHealth development is important to enable creating by evaluation [7,8,14,26].

**Figure 1:**
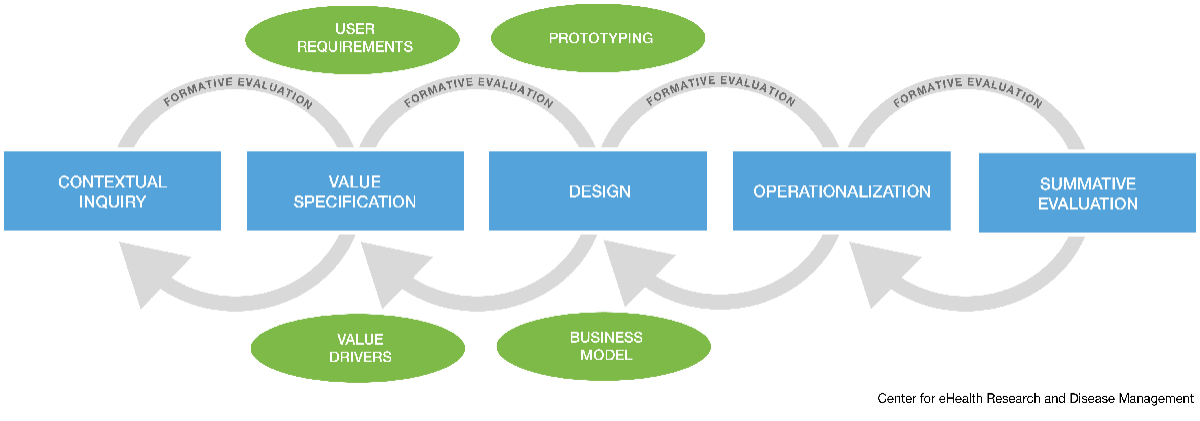
The CeHRes Roadmap (the Centre for eHealth Research Roadmap) [9].

These principles are translated into a model with 5 phases with accompanying goals, which are presented in Figure 1 [9]. This model can be used by developers to shape their development approach [3,6,9]. As the aim of this paper is to describe the development of eHealth technology and not the implementation or evaluation, the focus lies on the first 2 phases of the Roadmap: the contextual inquiry and value specification. These phases aim to create a thorough foundation for a technology and account for the interrelationship among the context, the people involved, and the technology. In the contextual inquiry, relevant stakeholders are identified, their roles, tasks, and opinions are analyzed, and the current situation and its weak and strong points are described to determine if and in what way technology can contribute. In the value specification, the values of the key stakeholders have to be identified and prioritized to determine what the added value of a technology should be. These values have to be translated into specific requirements that state what the technology should be able to do and look like [6].

### Case

Due to the involvement of 2 of the researchers in the development process, this research can be labeled as an action study. In this study, the development process of a VR application for the treatment of forensic psychiatric patients is presented. This project was initiated and mostly took place at Transfore, a forensic hospital in the east of the Netherlands, which offers forensic mental health care to both in- and outpatients. Forensic mental health care is a complex branch of mental health care, which is situated at the intersect between mental health care and the law because it deals with the combination of mental illness and delinquent behavior. In forensic mental health care, inpatients who reside in a closed setting and outpatients who are living at home are treated for sexual or aggressive criminal behavior [20,21]. A primary goal is to prevent criminal recidivism by means of treatment of offense-related factors, such as antisocial behavior or coping skills. Owing to their low motivation for treatment, low educational levels, and comorbid psychiatric disorders [22-24], forensic psychiatric patients can be characterized as a vulnerable patient population [25,26], which can be hard to include in research [27].

Multiple studies have pointed out the potential of VR for the assessment and treatment of forensic psychiatric patients [27-29]. VR offers the possibility to practice coping skills instead of talking about them, can be used to overcome practical issues for inpatients residing in clinics, and can enable therapists to observe patients’ reactions to offense-related stimuli or situations, such as children, drugs, or aggressive persons [29-31]. In VR, users enter computer-generated worlds that substitute their real-world sensory experiences with virtual ones [32], resulting in a feeling of presence: a sense of actually being in a virtual place [33]. Although VR applications have been used in mental health care, especially in exposure therapy for phobias [34], not much is known about its application in the treatment of forensic psychiatric patients [27]. Furthermore, little attention has been paid to how VR interventions should be developed for mental health care in general [32]. In our recent systematic review, we found that there are hardly any studies that discuss the development of technologies for forensic mental health [28]. However, especially in such a complex context in which there is little experience with the application of VR, thorough development is important [10,27]. Consequently, a thorough contextual inquiry and value specification to provide a good foundation for the application were especially important.

### Materials and Procedures

**Figure 2:**
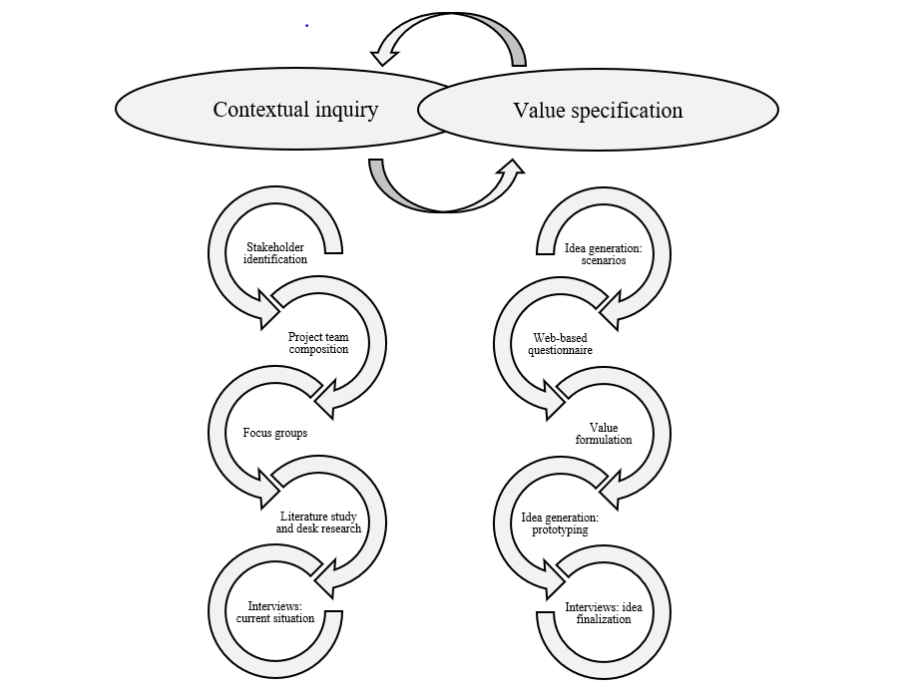
An overview of the used methods in the contextual inquiry and value specification phases of this study.

In this study, multiple methods were used to operationalize the first 2 phases of the CeHRes Roadmap. The development process started with the contextual inquiry. In this phase, the stakeholders were identified, a literature review was conducted, and a multidisciplinary project team to coordinate the project was constituted. Also, focus groups and interviews with forensic patients and therapists were held. In the value specification phase, 6 scenarios with concepts for VR applications were generated by the multidisciplinary project team. These concepts were presented to the patients, therapists, and stakeholders in a Web-based questionnaire. Next, values were formulated and used to create a concept for a VR app. This concept was visualized in a low-fidelity prototype and presented to the patients and therapists in an interview to examine their opinions and preferences. These activities were not performed sequentially: several methods were conducted alongside each other or were updated throughout the process [18]. Figure 2 provides an overview of the methods used in the development process. The arrows represent the iterative nature of the process and show that the methods and results of the contextual inquiry and value specification are not strictly separated but overlap. For more in-depth information about the results of the interviews and questionnaire, we refer readers to 2 other papers [29,30] that focus more on the content of the results and potential of VR for forensic mental health instead of a reflection on the methods and overall development process.

### Analysis

To reflect on the suitability of the methods and overall development process, we provided the most relevant information about each research method in a comprehensive table. The aim of this table is to present the goals, methods, results, and experiences with each method as clearly and concisely as possible. For each development method, the following information is reported:

- *Research question:* The research question for the development activity.
- *Method:* The name of the method, including the most relevant methodological information.
- *Target group:* If applicable, a description of the target group of which the data were collected and characteristics of the participants.
- *Main results*: A summary of the most important results and, if necessary, a reference to a Multimedia Appendix with further information about these results.
- *Conclusions:* The main conclusions and recommendations for further steps of the development process, which were drawn based on the results.
- *Lessons learned*: A reflection on the suitability of the method for the specific development phase, target group, and research question.

## Results

### Contextual Inquiry

In the contextual inquiry, we generated an overview of relevant stakeholders and their roles and tasks. Furthermore, the current situation and its points of improvement were analyzed to determine if and in what way VR could contribute to treatment in forensic mental health [6]. We used multiple methods that are provided in Table 1 below.

**Table 1:** An overview of the methods and outcomes and reflection on these methods of the contextual inquiry.

Research goal	Method	Target group	Main results	Conclusions	Lessons learned
Creating an overview of people and organizations who had a stake in the development process	*Stakeholder identification:* Via desk research, expert recommendations, and snowball sampling [31], constantly updated throughout the project	Not applicable	Identification of a broad range of stakeholders, such as end users, financers of care, knowledge institutes, and other forensic care organizations—see Multimedia Appendix 1 (1.1) for a visualization of the identified stakeholders	Stakeholder identification was useful to identify potential financers, participants, or institutions for data collection and to look for potential development partners	This method served well as a starting point for the project, but as in-depth information about (key) stakeholders was lacking, additional research into stakeholder perspectives was necessary, for example, via interviews. The stakeholder identification was constantly revised over the course of the project to keep it up-to-date. The identification proved to be important in preventing the relevant stakeholders from being overlooked in the development process and also in supporting the researchers in identifying participants for studies.
Constituting a multidisciplinary project team comprising patients, therapists, managers, and researchers to coordinate the project	*Project team composition:* In total, 5 potential end users (patients and therapists) were asked to join the team by the policy advisor of the organization in which the project took place (convenience sampling) to coordinate the project [6]. A total of 2 researchers were added for methodological and theoretical knowledge	Not applicable	The project team with 2 patients, 3 therapists, 2 researchers, and 1 policy advisor (n=8) was responsible for content-related and practical activities, such as structuring the development process, setting up studies, and accompanying research goals, interpreting results, decision making, and planning	The multidisciplinary project team was found to be essential for the coordination of the project, mostly because of the integration of different perspectives.	Including potential end users in the project team was useful to ensure that decisions were aligned with their perspective. In hindsight, the team might have benefited from someone with more technical knowledge on VR^a^, for example, a developer. Practical issues can influence the project team composition, for example, sometimes therapists or patients did not have enough time. It was important to make agreements on what to do when this occurred. Structure was needed to keep members involved: setting regular meetings, clear communication in between meetings, and keeping minutes of meetings. Coordination by a project manager was important to achieve this. The project team members had individual, concrete, and specific tasks that helped in keeping everyone actively involved. Patients indicated that participating in the project team gave them a sense of purpose and helped them with their treatment.
Determining how far there is support and enthusiasm for VR^a^ in forensic mental health care and identifying the ideas of therapists and patients about potential ways of using VR in treatment	*Focus groups:* Structure: presentations on VR by 2 companies, trying out VR by participants, individually coming up with ideas about VR in treatment, creating ideas in groups of 4, presenting the ideas to the entire group ; The duration of the focus groups was 2 hours and data were collected via researchers’ notes and templates filled in by the participants (see Multimedia Appendix 1: 1.2).	Patients (n=14) and therapists (n=23)	Most participants were very positive about VR. There was a broad range of ideas about using VR, for example, to improve skills, enhance insight by therapists or loved ones, or treat specific disorders, such as psychosis or posttraumatic stress disorder. See Multimedia Appendix 1 (1.3) for a table with the main results of the focus groups.	There appeared to be many possibilities, but further specification and insight into why and how VR should be used was required	Focus groups were a good and efficient way to start this broad, complex project with many possible outcomes, mostly to get an idea of attitudes and potential end users. These focus groups aimed to generate idea, so provided little in-depth information about needs and goals. It was necessary to complement them with other methods, such as interviews. The way this focus group was set up was seen as a strong point: there was a clear structure without much steering on content, which enabled all participants to brainstorm freely and individually. This resulted in a very broad range of ideas, which was relevant for this phase of the development process. It was relatively easy to find participants for the focus groups. An important reason for this seemed to be the possibility to learn more about and try out VR.
Gaining an overview of all studies and current initiatives concerning VR in treatment of (forensic) psychiatric patients	*Literature study and desk research:* Scientific database, search string (virtual reality OR VR OR augmented reality OR AR) AND (treatment OR intervention OR therapy) AND (forensic OR offend* OR crim*) and searching the internet, talking to stakeholders, and visiting conferences	Not applicable	In July 2017, only 6 relevant studies were found, mostly focused on the assessment of sexual delinquents [32-35] or general literature studies on VR [36,37]. Multiple ongoing projects were identified via desk research but with no accompanying scientific publications or available products	Not much is known about VR in forensic mental health care in both practice and research, so there appeared to be a need for a bottom-up development process to identify why and in what way VR could be used	Especially, desk research proved to be relevant for the project because there were no publications (yet) about many recent, ongoing initiatives/projects. The strategy for desk research could have been more structured, for example, by creating an activity plan and planning recent updates of desk research. It was important to look outside of the focus of the project (eg, studies on VR in general), either by conducting a literature study (which is time consuming) or by searching for published reviews or meta-analyses. It might have been useful to systematically collect the literature on theories and models on delinquent behavior, as in this project, it was done in a more ad hoc manner.
Identifying points of improvement in the existing forensic mental health treatment of in- and outpatients and possible applications of VR, which could improve the current situation, according to therapists and patients	*Interviews: current situation:* The first part of the interview scheme focused on points of improvement of the current treatment (regardless of VR) [21], the second part focused on the possibilities of VR to improve the current treatment. The outcomes of the focus groups were used to structure the interview scheme	Therapists (n=8) and patients (n=3), working or treated at multiple locations of Transfore, the forensic hospital.	Via inductive coding [36], 2 types of codes were identified in line with the 2 research questions. Points of improvement were related to patients’ return to society; specific patient characteristics, such as treatment motivation; and treatment characteristics, such as skills training. Possibilities of VR were skills training with interaction, observation of patients’ reactions, and creating insight for others. The codes can be found in Multimedia Appendix 1 (1.4)	The interviews gave much information about why and how VR could be of added value. However, there were still too many possible directions to make a grounded decision about the goal and content of VR. Additional research into the needs and wishes of end users was required	The participants were asked to provide scenarios about their own experiences and ideas in an open, explorative manner to prevent too much steering by the researchers. To gain in-depth information, good interviewing skills and probing questions appeared to be important. Eliciting scenarios in participants proved to be unsuitable for (most) patients, mostly because of the broad questions that required much abstract reasoning. The part with examples from the focus groups worked better but was still experienced as difficult. Also, the interview took 1 hour, which proved to be a threshold for participating and resulted in difficulties with inclusion. The type of information collected via the interviews would have been hard to retrieve via questionnaires because of the need for probing questions. The research questions might have also been answered by means of (small) in-depth focus groups, which might have been less time-consuming.

^a^VR: virtual reality.

### Value Specification

In the value specification phase, the outcomes of the contextual inquiry were used to further specify what the added value of a technology should be according to the key stakeholders. Again, multiple methods were used to identify the stakeholders’ preferences and opinions on VR in forensic treatment and prototypes to specify these abstract values were created. These methods are provided in Table 2.

**Table 2:** An overview of the methods and outcomes and reflection on these methods of the value specification.

Research goal	Method	Target group	Main results	Conclusions	Lessons learned
Generating multiple ideas on the use of VR^a^ in forensic mental health care, based on the outcomes of the contextual inquiry	*Idea generation—scenarios:* In 3 sessions, all project team members individually brainstormed about ideas for VR applications. The 6 most promising ideas were worked out in a template (see Multimedia Appendix 1: 1.5) by multiple project team members. On the basis of these templates, scripts were written and 6 short videos were filmed	Not applicable	A short video was created for each of the 6 ideas. All videos had the same underlying structure: the goal of VR, its use during treatment, an example, and the desired outcomes. The videos (with English subtitles) can be watched on YouTube [38]. An example of a scenario can be found in Multimedia Appendix 1 (1.6)	The videos made clear that there are a lot of promising possibilities for VR in forensic mental health, so it appeared to be necessary to make decisions about what to prioritize and why	The structured approach in which multiple templates were used worked well in this project: it forced all different members of the project team to work and think in a similar way. Each member of the project team had a clear role with individual responsibilities. This was experienced as helpful in motivating the team members and ensuring that all of their perspectives were present in the 6 ideas. Creating scripts and videos was very time-consuming, so motivated members who are willing to invest time and effort and enough budget were necessary preconditions for making videos.
Identifying (1) the preferences of stakeholders of the 6 ideas and (2) the stakeholders’ values regarding VR in forensic mental health care	*Web-based questionnaire:* After asking sociodemographic questions, the 6 videos were presented to the participants in random order. After each video, the PII^b^ [39,40] a question about the participant’s grade for the idea, and 3 open questions on positive points, points of improvement, and suggestions for the idea were provided	Patients (n=19); therapists (n=89); other stakeholders (n=38), such as parole officers or researchers from different Dutch forensic institutions	There were no significant differences between the grades and PII scores for ideas. A broad range of positive and negative aspects and remarks were identified via inductive coding. These can be found in Multimedia Appendix 1 (1.7)	The results of the questionnaire were mostly in line with the interviews but provided more detailed and specific information, for example, how VR should be personalized and which skills should be trained	The answers of the patients fitted the research questions of the questionnaire better than the answers that were given by patients in the interviews. This indicated that the concrete, scenario-based videos were a better way to include the patient perspective than the broad, abstract interviews. Although the goal was to make this method less time-consuming, filling in the questionnaire still took about 30 minutes, which might explain why a large share of the participants (55.4%) did not fully complete it. A shorter questionnaire might have led to more response but also would mean that less information would have been retrieved. The quantitative measures indicated no major differences between opinions about ideas. Although it was not clear if this was an issue regarding validity or if there actually were no differences, it was still useful to ask for a grade for each idea. The PII was not of added value in this questionnaire. Although this method proved to be useful to further specify previously found results, it would not have been suitable as an initial method to gather in-depth information, partly because no probing questions could be asked, and answers were relatively short.
Formulating values that capture what the added value of the technology should be for people and context, according to the stakeholders	*Value formulation:* On the basis of all previous results, 2 researchers created attributes that summarized the needs or wishes of stakeholders [17]. On the basis of categories of related attributes, accompanying values that stated what VR should achieve, improve, or add according to the stakeholders [6,17] were formulated. The values were discussed by the project team and minor adjustments were made accordingly	Not applicable	A total of 43 attributes and 13 values were formulated. An example of how a value was created can be found in Multimedia Appendix 1 (1.8). The following values were formulated: fit with patient; improvement of skills; insights into behavior, thoughts, and feelings; bridge between treatment room and practice; generalization of skills to daily life; safety; treatment motivation; unique addition to current treatment; ease of use within treatment; cooperation between patient and therapist; wide applicability; affordability; and constant adaptation of the application	Formulating values proved to be a very good way to *get to the point* and summarize the essence of the results so far. It forced the project team to critically think about the overall added value and goals of the VR app and prevented them from getting lost in details or a tunnel vision	Values might be difficult to understand for outsiders as they are abstract, concise summaries of the needs and wishes. Consequently, clear definitions of the values were provided to prevent misunderstandings. Besides their importance for development, the project team determined that values could also be useful to determine what to evaluate: to what extent was the added value actually achieved in practice? This way of thinking about values allowed the project team to think ahead in terms of implementation and evaluation and facilitated a broader view on the VR application. In hindsight, the process of formulating values was more complex than expected. The project team had to account for the results of all used research methods, combine them in an abstract way, and make decisions about conflicting values, such as the importance of visual realism. A clear guideline for formulating values would have been useful.
Generating a concept for a VR application based on the values and previously gathered results	*Idea generation—prototyping:* The project team discussed the values, attributes, and outcomes of all research activities and their implications for a VR application. Via multiple brainstorming sessions in which multiple low-fidelity prototypes were created, a first version of an idea was developed	Not applicable	The main goal of the VR application was to support therapists and patients in identifying *triggers* that can elicit undesired behavior and search for *helpers* that can support the patient in dealing with these triggers. Patient and therapist can together build personalized scenarios via a dashboard with several categories that contain elements that can be added to a scenario (see Multimedia Appendix 1: 1.9 for the prototype)	The developed concept was a combination of elements of all 6 videos that were created by the project team. Also, important concepts that already arose from the interviews were present in the idea, for example, personalization, skills training, and new insights	To ensure the consistency of the development process, the idea generation process started with discussing the implications of all earlier conducted studies, even though it was more appealing for the project team to start creating the idea right away. Visualization of ideas via low fidelity (lo-fi) prototypes appeared to work well during the idea generation process to make abstract concepts more concrete. For example, the team drew multiple dashboards and visualized the structure of the dashboard with post-its. This was experienced as helpful by all members of the project team.
Investigating (1) how far the stakeholders’ opinions of the concept match the previously formulated values and (2) if changes to the concept are required for it to optimally fit the stakeholders’ preferences	*Interviews—idea finalization:* In the first part, open-ended questions, based on an adapted version of the TAM^c^ [41], were asked to check the attitudes toward the concept of the VR application. The second part focused on the participant’s overall opinion of the VR application. The developed low-fidelity prototype and a scenario on its use in treatment were used	Patients (n=10) and therapists (n=8) from all different locations of Transfore, the forensic hospital	The first part was coded deductively using the constructs of the TAM (see Multimedia Appendix 1: 1.10), the second part was coded deductively with the 13 formulated values (see Multimedia Appendix 1: 1.11). Overall, the idea was evaluated positively, but there were some concerns about the ease of use of the application. All values were, to some extent, present in the participants’ answers. Most positive remarks were about the added value for treatment, for example, fit with patient and new insights. Points of attention were related to the implementation in treatment	Overall, the idea fits the values of the participant, mostly with regard to the unique added value to treatment. No major changes to the basic idea were necessary. In later stages, attention should be paid to the usability of the application, training, and protocols to successfully embed VR in treatment	This second set of interviews was considerably shorter than the first one: they only took about 15 to 20 minutes. It proved to be easier to include patients, which might be because of the relatively little time that was required to participate. Using the values to code these interviews was useful to determine the positive and negative aspects of the idea in relation to the added value that it should have had. In this way, it became very clear what the points of improvements were, which might not have been the case with an inductive, bottom-up coding process. It also allowed the project team to check whether the idea was still in line with the values. The TAM was used in the interview scheme and coding process. Although it helped to structurally ask about and analyze the participants’ attitudes and intentions, it provided hardly any information about the treatment context and characteristics of (other) persons [42,43]. The second part, in which the added value in general was discussed, appeared to be necessary to paint a full picture of the participants’ opinion. Merely using the TAM would not have sufficed in this interview.

^a^VR: virtual reality.

^b^PII: personal involvement inventory.

^c^TAM: technology acceptance model.

## Discussion

### Reflection on Development Methods

The main goals of this study were to analyze the suitability of the development methods for participatory eHealth development in a complex context and reflect on the development model used: the CeHRes Roadmap. This study can contribute to the development of a broad tool kit from which researchers can choose appropriate methods for the stage of their development process, participants, and context. In hindsight, this study would have benefited from such a tool kit, as the results showed that all methods generated valuable information, but not each method proved to be very suitable for the target group and their context. Besides generating knowledge on suitable methods, this type of study can also facilitate reflection and accompanying improvements of the development model used. Although this study offers a contribution, more studies that pay attention to development methods and models are required to make generalizable statements about methods and models.

The first goal of this study was to reflect on the suitability of different development methods. The relevance of this goal became clear from the experiences of the project team, as a major challenge was to identify the suitable methods for the forensic psychiatric patient population. These types of vulnerable patient populations are often difficult to involve in research, and not much is known about the suitable methods for these types of population [27,44]. On the basis of the experiences with methods used in this study, several conclusions and recommendations can be drawn on the suitability of methods.

A first set of recommendations focuses on involving patients in research. First of all, working with concrete examples seemed to work better than merely asking patients for their opinion or ideas without much guidance or input [4]. Using existing or potential examples is also possible in the earliest stages of the process, when not much is known yet, and can be done by using methods derived from a human-centered design, such as scenarios, personas, or prototypes [45,46]. A second recommendation based on the findings of this study is to keep data collection as short as possible, because patients might have difficulties with concentration or are not motivated to invest a lot of time. This recommendation is also relevant for health care professionals, because although researchers often want to collect as much data as possible, the professionals often not have a lot of time to participate [17]. The balance between how much in-depth information should be collected and the duration of data collection was experienced as difficult, so more research on this topic is needed. Finally, participating in research should be perceived as personally relevant or rewarding [47]. Although we used rewards such as VR goggles in the questionnaire and interviews, including participants for the focus groups proved to be easier. A reason for this might have been that participants could experience VR during the focus groups, which was perceived as new and exciting by both patients and therapists. Consequently, it appears to be worthwhile to spend time on identifying personally relevant rewards for participants.

The second set of recommendations centers on combining multiple methods and perspectives to paint a clear and complete picture of the context and stakeholder perspective. First, although involving patients proved to be very valuable, the development process also benefited from the perspectives of other types of stakeholders, such as therapists, managers, researchers, and technology developers, as they might have different needs or a more overarching view [28,48]. For example, the analysis of the first set of interviews showed that patients mostly mentioned the use of VR to observe situations and stimuli, whereas therapists also pointed out the importance of other possibilities, such as skill training, which was not mentioned by the patients. Second, involving participants via multiple methods enabled the project team to gain different types of information that supported them in getting a good grasp of all perspectives on VR in forensic mental health care. Finally, it can be concluded that more knowledge on suitable methods for involving patients, therapists, and other stakeholders in eHealth development is needed to be able to make more generalizable statements and create a tool kit [47].

### Operationalization of the Development Model

Besides reflections on development methods, this study also aimed to reflect on the application of the development model that was used: the CeHRes Roadmap. It is of course not possible to conclude whether the development process guided by the CeHRes Roadmap resulted in better outcomes than another development method, partly because that would require 2 parallel development processes in identical settings [18], which is difficult both practically and conceptually. Nevertheless, based on the experiences of the project team, it can be concluded that the CeHRes Roadmap provided a valuable guidance for the development process. This process resulted in a concept for a VR application that is based on the wishes and preferences of the therapists and patients. The fit with their wishes became especially clear in the second round of interviews that showed that participants were enthusiastic about the concept and their opinions closely matched the previously formulated values.

On the basis of the experiences of this study, several recommendations can be made on how to operationalize the CeHRes Roadmap and similar development models. First of all, an important principle of eHealth development is that it should comprise multiple formative evaluation cycles. The experiences of this study confirmed that the Roadmap should not be used as a linear, sequential approach with a fixed order of phases and accompanying activities [18]. To illustrate, the first set of interviews and focus groups provided information that was relevant for both the contextual inquiry and value specification phase. Also, during the value specification, activities from the design phase, such as prototyping and scenarios, were used to elicit opinions. Consequently, although the phases of the Roadmap are visualized as separate blocks (see Figure 1), they should be used as overlapping, interwoven sets of principles and methodologies. A thorough understanding of the principles of the Roadmap appeared to be more important than strictly following the order of separate phases.

**Figure 3:**
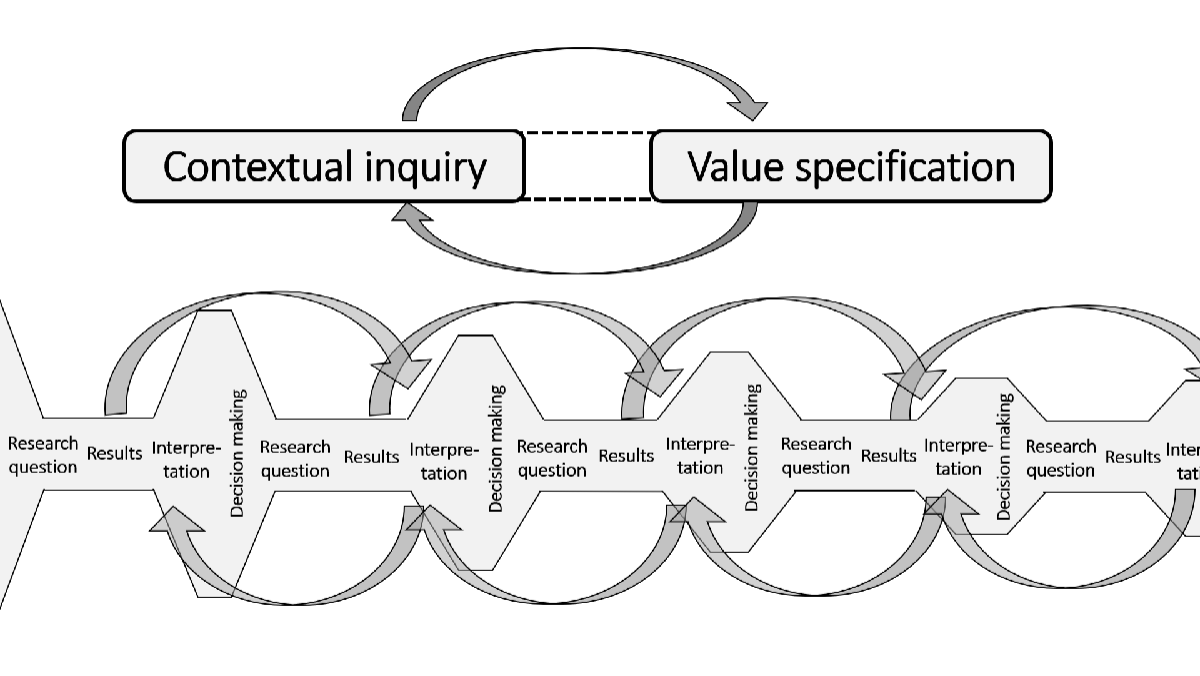
The structure of the goal-driven development process with multiple formative evaluation cycles.

A second important finding was that the formulation of clear, specific research goals was pivotal in structuring this development process. A pitfall of an elaborate development process in a complex setting is that it might become unstructured or vague [6,14]. We tried to prevent this by formulating multiple clear, specific research questions that were based on the goals of the Roadmap’s phases and its 5 underlying principles [6]. To keep the process coherent, the project team carefully thought about how these research questions related to the outcomes of the previous development activities. Also, we added multiple formative evaluations to check whether the outcomes of different activities remained consistent with each other. This process is visualized in Figure 3.

Third, although constituting and managing an interdisciplinary project team was complex and time-consuming, the team was found to be an important part of the development process as it facilitated decision making from multiple perspectives [22,49,50]. Multidisciplinary teamwork in health care is often complex [51], so several measures were taken to increase the chances on a successful collaboration. Among other things, patients and therapists that participated in the project team were involved as active co-designers instead of passive informants [52,53] and thus took part in activities, such as designing studies, interpreting results, and creating and adapting ideas. To achieve this, the project leader ensured that each project team member had a clear task, as was, for example, done in the creation of scenarios, where each member actively participated in creating an idea and writing the script for 2 of the videos. Fourth, much attention was paid to the functioning of the team. Among other things, roles and tasks of all team members were made clear; regular, bimonthly meetings were held and there was ample communication in between meetings; individual members got the opportunity to be involved in activities of their own choice; there was a mix of skills and interests of members; there was a positive climate of trust and common respect; and, importantly, the team had a common, clear goal [51]. However, as these findings are based on only 1 development process, they are not generalizable. As the functioning of a project team seems to be a relevant topic in eHealth development, more studies on how to compose and organize multidisciplinary project teams should be conducted to be able to draw generalizable conclusions and recommendations. Finally, when operationalizing the CeHRes Roadmap—or any other development model—a thorough understanding of the model’s underlying principles, continuous formative evaluations to prevent tunnel vision, clear research questions with suitable methods, and a well-functioning multidisciplinary team were found to be important.

### Reflection on the CeHRes Roadmap

While using the CeHRes Roadmap to shape the development process, we identified several strong points but also some points of improvement. First of all, the participatory development principle was used to determine what the main goal of the VR application should be in a bottom-up manner. According to this principle, it is important to involve users from the start to ensure that a technology addresses actual problems or points of improvement and is of added value for them [54]. However, in many cases, the goal of an eHealth technology is determined by researchers and/or developers, and stakeholders are involved as mere informants in later stages to provide feedback on concepts that were created in a top-down manner [55]. In this project, we tried to prevent this by actively involving stakeholders from the start, among other things, by asking them about points of improvement of the current situation and enabling them to come up with their own ideas about VR. Further along the process, values were formulated to specify the goal of the VR application. These values forced the project team to explicitly state the added value that a technology should have for patients and therapists. However, during the value specification, we noticed that there was a lack of clear guidelines on how to formulate these values and what topics they should cover. Although this value-driven approach was experienced as useful to keep an eye on people and their context, there is still much uncharted territory. We recommend that more studies using values in their development process should be conducted to be able to create clear guidelines.

Second, the Roadmap emphasizes the importance of formative evaluation and use of multiple methods. This indeed proved to be essential in this development process, especially because at the start of the project, there was no knowledge about the use of VR in forensic treatment. Consequently, much information had to be generated to make substantiated choices for the goal and content of the VR app. Just using 1 or 2 research methods would not have sufficed. This can be illustrated by the following example on personalization of VR. The first interviews and literature study indeed pointed out that personalization was important [35-37] but did not provide in-depth information about this topic. The results of the questionnaire offered more insights into what stakeholders wanted to be able to personalize: virtual people, environments, and scenarios. Throughout the process, the project team further specified these preferences and translated them into concepts for personalized VR applications via low-fidelity prototypes that were evaluated with stakeholders and fine-tuned accordingly. If only 1 interview study would have been conducted, the project team would not have had enough input to create a personalized VR application. A disadvantage of the multimethod, iterative approach was that it was very time-consuming. It might be possible that, if more would have been known about VR in forensic mental health care or suitable development methods, less research would have been required, which might have resulted in a shorter and more efficient development process. But again, more research on different types of development methods is required to draw more conclusions on this topic.

Finally, when reflecting on the development process, a more systematic approach toward involving domain-specific theories and models could have been used. Owing to the involvement of researchers and professionals with much knowledge on existing treatment models and theories on offending, this information was included but in an ad hoc manner. As other studies and models such as intervention mapping point out, it is important to incorporate theories that explain and change behavior in eHealth interventions [7,12,56,57]. In this project, this relates to models that explain delinquent behavior or theories that underpin treatment of forensic psychiatric patients, such as the general theory of crime [58] or the risk-need-responsivity model [59]. Consequently, we recommend that the use of domain-specific theories and models to explain behavior and treatment can be explicitly integrated in the Roadmap. To do this, the pillar on persuasive design could be adapted. In its current state, it focuses on behavior change via persuasive design. We suggest a change to this pillar, so that, besides persuasive theory, it also entails the use of domain-specific theories and models throughout the entire development process. Goals and activities derived from this adapted principle could be added to the contextual inquiry and value specification phases to add more focus on domain-specific theories at the beginning of the development process.

### Conclusions

This study described and reflected on the methods and development model used in a development process of a VR application for a complex setting: forensic mental health care. To take the domain of eHealth development to the next level, more studies need to report and reflect on the development processes in a standardized way to generate more knowledge on suitable methods. This might result in a tool kit that researchers can use to choose and operationalize methods. Based on this study, we conclude that eHealth development is much more than programing a technology or just going with the flow; it requires thorough research via methods that fit the participants, stage in the development process and context, structured project coordination by a multidisciplinary project team, a flexible and open mind-set, and the inclusion of multiple perspectives in every decision.

## Appendix

Multimedia Appendix 1 Main results of the research methods.

## Abbreviations

CeHRes: Centre for eHealth Research
eHealth: electronic health
PII: personal involvement inventory
TAM: technology acceptance model
VR: virtual reality
