# Supplementary material for: The Importance of Systematically Reporting and Reflecting on eHealth Development: Participatory Development Process of a Virtual Reality Application for Forensic Mental Health Care
Source: J Med Internet Res. 2019 Aug 19;21(8):e12972. doi: 10.2196/12972 (PMC6718085; doi:10.2196/12972)
Supplement: Multimedia Appendix 1 [file jmir_v21i8e12972_app1.pdf]

Multimedia Appendix 1 – Main results of the research methods

Appendix 1.1 - A visualization of the identified stakeholders

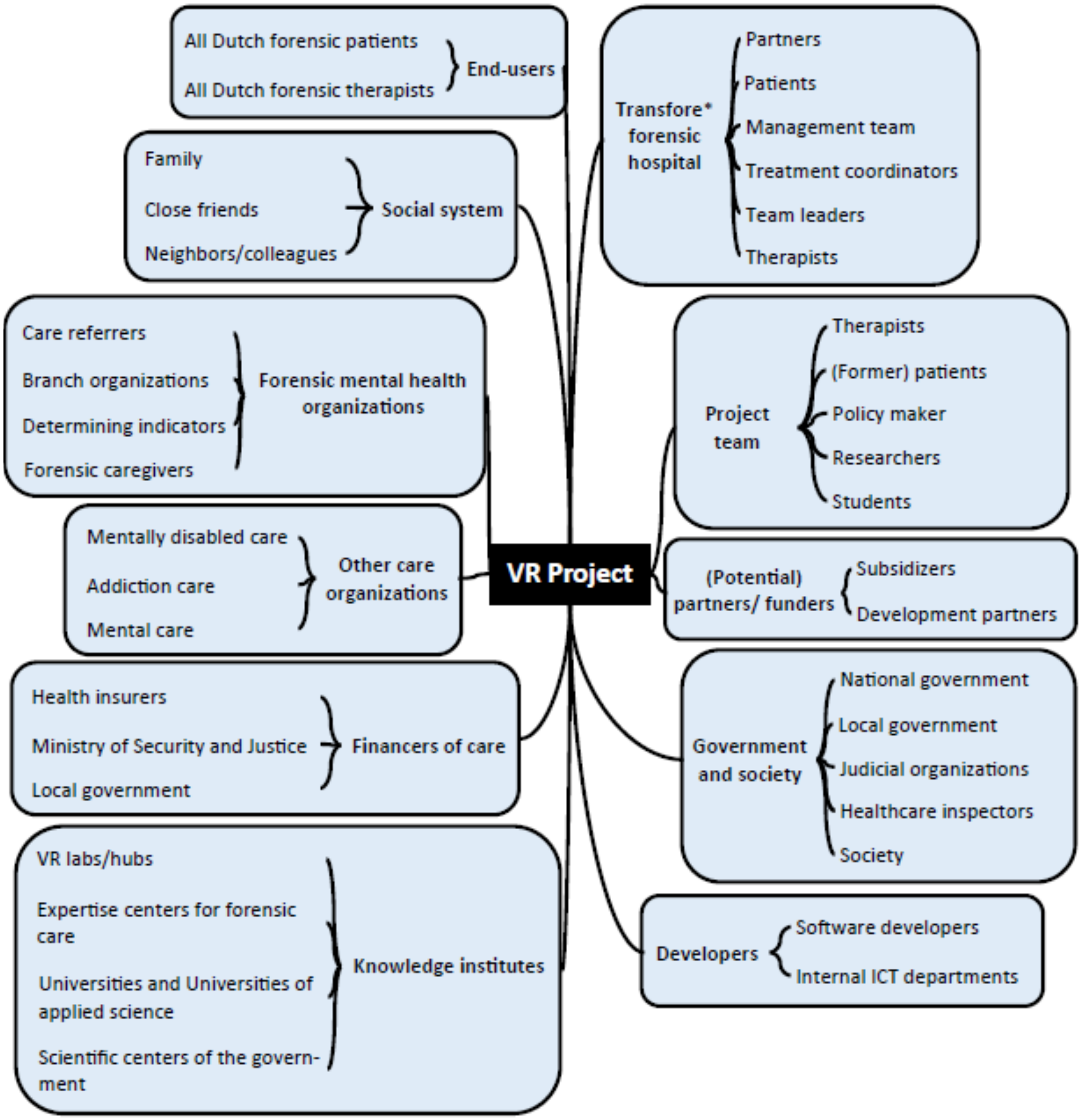

**Transfore is the forensic hospital in which the current project was initiated and mostly took place.*

Figure 1.1. An abridgement of the stakeholder identification

Appendix 1.2 – Template used in the focus groups

Table 1.1. The template that participants in the focus groups filled in.

How could virtual reality support you or other people in treatment at Transfore?		
For who? What's the target group?	What should it look like?	What could one learn from it?

Appendix 1.3 - The main results of the focus groups

Table 1.2. The four main categories and accompanying sub categories of the focus group's outcomes.

Skill	Insight	Treatment of disorder	Design
Daily living skills<br>Skills related to offense	Patient observes own behaviour from other's perspective<br>The other person observes patient's perspective<br>Delinquent behaviour in realistic situation	Anxiety<br>Psychosis<br>Depression<br>Autism	Importance of realism<br>Personalization

Appendix 1.4 - The main results of the interview study of the current situation

Table 1.3. Points of improvements and possibilities of VR according to therapists (Ther.; n=8) and patients (Pat.; n=3), and the total number of interviews in which the code was identified (Tot.)

Main and sub codes	Total	Thera-pists (n=8)	Patients (n=3)
**<u>Point of improvements of the current treatment</u>**			
**Characteristics of the forensic setting**			
Return to society – emotions and cognitions	**3**	3	
Return to society – skills	**4**	4	
Return to society - recidivism	**6**	5	1
**Patient characteristics**			
Treatment motivation	**4**	4	
Patients' low educational level	**4**	4	
Emotion regulation	**5**	5	
**Treatment characteristics**			
Skills training in context	**7**	6	1
Therapist's insight in patient	**5**	4	1
Exposure therapy	**3**	3	
Physical activity	**2**	2	
Involvement of significant others	**3**	2	1
**<u>Possibilities of VR to improve current treatment</u>**			
**Skills training with interaction**			
Training daily living skills	**5**	5	
Training social skills	**6**	5	1
Training emotion regulation skills	**9**	8	1
**Observing without interaction**			
Exposure to emotion-eliciting stimuli	**9**	6	3
Observing daily life	**2**	1	1
Observing behaviour	**8**	6	2
**Creating insight for others**			
Insight into reactions to triggers	**5**	5	
Insight into patient's situation	**7**	5	2

Appendix 1.5 – Template for the development of video scenarios used by the members of the project team

**Template for development of video scenarios**
**Part 1: Underlying concept**
Please describe here the core of the VR idea in a way that is understandable for (almost) all therapists and patients. This core should be based on one of the six concepts that were created by the project team in the last meeting. This text will be used in the questionnaire, so try to keep it as clear and concise as possible. This part should really focus on the abstract concept behind the VR application, so don't give any examples yet.
**Part 2: Concrete explanation**
The second part of the presentation of the concept consists of a short video in which an example of a VR application is presented to illustrate the previously described underlying concept. Please describe here, for the project team, briefly the idea that you will be presenting in the video. You can do this via written text, since this part will serve as background information for the project team and will not be used in the questionnaire.
**Part 3: Elaboration of video script**
Please add a draft script of the video here, if possible, with drawings, pictures or photo's to further clarify. You can do this via a storyboard from which the storyline and design of the video become apparent. It should become clear what type video it will become (e.g. animation, Claymation, 'real' film) and what the content is. Also think about matters as whether or not spoken text is necessary, what the voice-over text should be, and how long the video will approximately take. Try to provide as much detail on the final video.

Appendix 1.6 - An example of a scenario with screenshots of the video

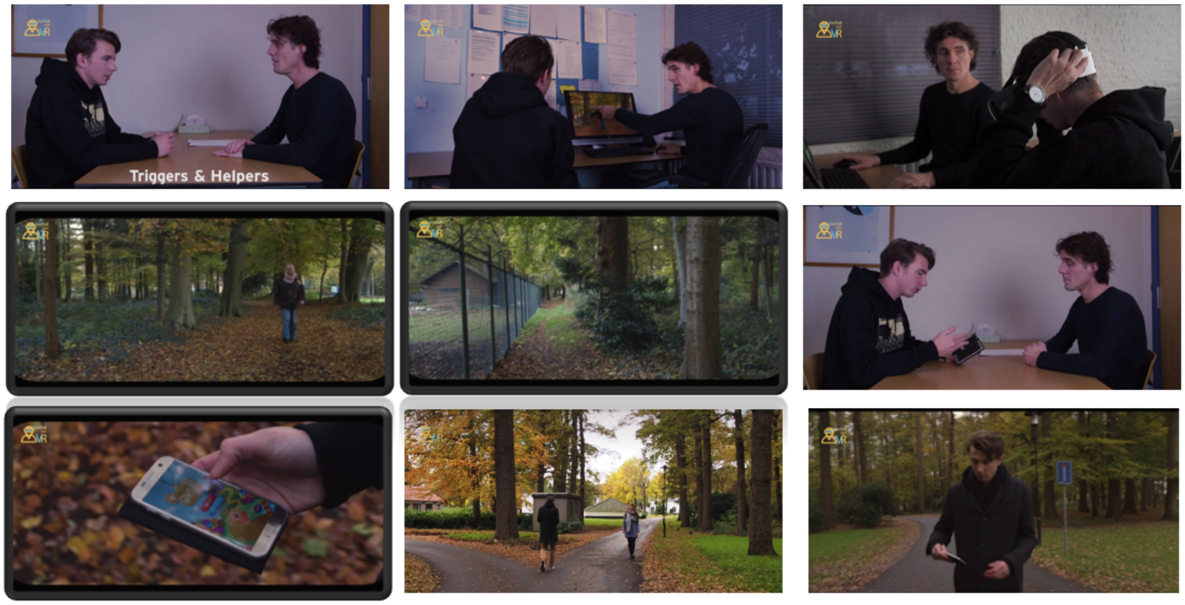

Figure 1.2. Screenshots from video 1, 'Triggers & helpers': a patient and therapist are discussing difficult situations and associated triggers, in this case running into a woman. The patient is exposed to this situation in VR and afterwards, patient and therapist are discussing what might help him in dealing with the trigger. The patient practices with using a phone as a helper in VR, and the video ends with a real-life situation in which the patient has transferred the skill and actually uses his phone as a helper.

Appendix 1.7 - The main results of the questionnaire study

Table 1.4. The codes of the answers to the three open questions, structured by the five main codes that were identified in all three questions.

Judgement	Therapist/ treatment	Patient	Content	Practical
		**Positive remarks**		
Positive judgement without explanation	Good addition to therapy; safe practicing; good exercise; insight into behaviour patient; cause for conversation; good addition to current treatment	Improvement (future) behaviour; insight own behaviour; insight behaviour others; treatment motivation; suitable for specific target group; supporting in reliving	Realism of behaviour; personalisation of persons, scenario & environment	Visual realism; new, different technology
		**Negative remarks**		
Negative judgement without substantiation	Not or difficult to use in treatment; not of added value to current treatment	Dishonesty about effect; dishonesty about input scenario; not suitable for specific group; no effect; elicitation negative feelings	Not generalizable to real life; important element is lacking; no realistic behaviour; use of VR is not necessary to reach same goal	No visual realism; hard to use; costs; takes time during treatment; takes time to learn to use
		**Suggestions**		
Negative or positive recommendation about introduction in practice	Apply for specific type of treatment; good training of therapist; input for VR scenario should be well-prepared	Check whether VR is suitable for patient; co-creation of scenario with patient; apply to specific target group	Combine ideas; use realistic situations; change an aspect of the application	Combine with other technologies; improve visual realism; offer plenty choices in persons, scenarios & environments; context use VR; posture use VR; constant development

Appendix 1.8 - An example of how a value was created

Table 1.5. An example of a table with foundations from earlier research, attributes and a value.

Foundations	Attributes	Value
**Codes interviews**<br>Training daily living skills; Training social skills; Training emotion regulation skills; Skills training in context<br>**Questionnaire**<br>Good exercise; Safe practicing; Improvement (future) behaviour<br>**Literature**<br>Fromberger, Jordan & Muller (2014); Renaud et al. (2014)<br>**Project team**<br>Minutes meeting 8 & 10	Stakeholders think it is important that…<br>• the patient can acquire or improve daily living skills<br>• the patient can acquire or improve social skills<br>• the patient can acquire or improve emotion regulation skills<br>• interaction with a virtual other, played by the therapist, is possible<br>• behaviour of virtual others is realistic	Improvement of skills

Appendix 1.9 - Low fidelity prototype of the dashboard for the VR application

1. Environment			2. Virtual avatar of other person				3. Stimuli to add to scenario	
**1.1 Type of environment**	**1.2 Background sound (constantly present)**	**1.3 Extra(s)**	**2.1 Basic appearance**	**2.2 Physical appearance**	**2.3 Facial expression***	**2.4 Posture and gestures**	**3.1 Auditory stimuli**	**3.2 Visual stimuli**
Pedestrian street during night-time	Buzzing of people	Drug dealer with small (bum) bag	Male/female	Length (*enlarging/ reducing*) *	Happy*	Punching gesture	Ringing phone	Game on own phone
Pedestrian street during daytime	Loud talking	Large crowd of people (>10)	Age (6-80) *	Physique (skinny build/ broadly build) *	Scared*	Stopping gesture (hands forward)	Loud music	Bottles of alcohol/beer
Train	Sound of playing children	Loitering adolescents		Ethnicity (white/*north-African/African*)	Neutral*	Arms crossed	Music by own choice	One building that can be changed into coffee shop, liquor store, brothel or pharmacy
Living room	Background music	Playing children		Proximity (far away, *nearby*) *	Angry*	Anxious gestures: torso is moving, wobbling	Police sirens	Police car
Generic desk	Volume of sound (decibel)*	Someone observing the conversation of patient and avatar		Clothing woman *Normal sweater / short dress with cleavage / headscarf / top with panther print, large golden earrings, tattoo*	Sad *	Arms alongside the body	Crying baby	Sign with text that can be adapted/entered (e.g.: count to ten)
Park with fountain	Hertz of sound*	Two (attractive) females			Ashamed*	Shrinking movement/making posture smaller, arched shoulders	Screaming people	
Supermarket		Several random people spread out over location		Clothing man *Normal sweater or shirt / suit / tracksuit / wife beater or tank top with bare arms and tattoo*		Puffed out chest	Barking dogs	
Car		People drinking alcohol				Wild arm gestures	Two fighting people	
Forest		Two broad/strong-looking men				Giving the middle finger	Loudly ticking clock	
		Two skinny/weak-looking men				Kicking movement		
		Two officers				Tilted head (up or down)		
		Family of four				Clenched fists		
		Men walking while holding hands				Pointing		
		Group of police officers (both male and female)						

Figure 1.3. A lo-fi prototype of the dashboard for personalization of the VR application. The asterisks represent mean that this part of the dashboard is 'adaptable': it's a scale instead of an 'on or off' option.

Appendix 1.10 - The main results of the attitude-part of the idea finalization interviews

Table 1.6. The number of patients and therapists that positively mentioned one of the four constructs of the TAM

	Positive attitude Towards idea	Perceived potential usefulness	Perceived potential ease of use	Positive intention to use
Patients (n= 10)	9	9	6	5
Therapists (n =8)	8	8	5	8
Total (n=18)	17	17	11	13

Appendix 1.11 - The main results of the value-part of the idea finalization interviews

Table 1.7. The number of interviews with therapists (n=8) and patients (n=10) that contained potential valuable aspects or points of attention related to a specific value

Values	Potential valuable aspects (positive)			Points of attention (negative)		
	**Total (n=18)**	**Ther. (n=8)**	**Pat. (n=10)**	**Total (n=18)**	**Ther. (n=8)**	**Pat. (n=10)**
**Aim application**						
Fit with patient	**16**	8	8	**4**	3	1
Bridge between therapy room and practice	**14**	7	7	**4**	2	2
Insight into behaviour, thoughts and feelings	**14**	8	6	**2**	1	1
Improvement of skills	**7**	5	2	**0**	0	0
Generalization of skills to daily life	**5**	4	1	**2**	1	1
Safety	**1**	1	0	**5**	2	3
Treatment motivation	**1**	0	1	**6**	2	4
**Embedment in treatment**						
Unique addition to current treatment	**13**	6	7	**1**	0	1
Cooperation of patient and therapist	**11**	6	5	**2**	1	1
Content fits well with current treatment	**6**	5	1	**2**	2	0
Practically easy to use in current treatment	**5**	2	3	**8**	7	1
**Use application in practice**						
Continuous adaptation of application	**5**	3	2	**0**	0	0
Widely applicable	**1**	1	0	**9**	4	5
Affordability	**0**	0	0	**2**	1	1
